# Identifying sources of bias when testing three available algorithms for quantifying white matter lesions: BIANCA, LPA and LGA

**DOI:** 10.1007/s11357-024-01306-w

**Published:** 2024-08-08

**Authors:** Tatiana Miller, Nora Bittner, Susanne Moebus, Svenja Caspers

**Affiliations:** 1 Institute for Anatomy I, Medical Faculty & University Hospital Düsseldorf, Heinrich Heine University, Düsseldorf, Germany; 2 Institute of Neuroscience and Medicine (INM-1), Research Centre Jülich, Jülich, Germany; 3 Institute for Urban Public Health, University Hospital Essen and University Duisburg-Essen, Essen, Germany

**Keywords:** White matter lesion, BIANCA, LPA, LGA, Training data characteristics

## Abstract

Brain magnetic resonance imaging frequently reveals white matter lesions (WMLs) in older adults. They are often associated with cognitive impairment and risk of dementia. Given the continuous search for the optimal segmentation algorithm, we broke down this question by exploring whether the output of algorithms frequently used might be biased by the presence of different influencing factors. We studied the impact of age, sex, blood glucose levels, diabetes, systolic blood pressure and hypertension on automatic WML segmentation algorithms. We evaluated three widely used algorithms (BIANCA, LPA and LGA) using the population-based 1000BRAINS cohort (*N* = 1166, aged 18–87, 523 females, 643 males). We analysed two main aspects. Firstly, we examined whether training data (TD) characteristics influenced WML estimations, assessing the impact of relevant factors in the TD. Secondly, algorithm’s output and performance within selected subgroups defined by these factors were assessed. Results revealed that BIANCA’s WML estimations are influenced by the characteristics present in the TD. LPA and LGA consistently provided lower WML estimations compared to BIANCA’s output when tested on participants under 67 years of age without risk cardiovascular factors. Notably, LPA and LGA showed reduced accuracy for these participants. However, LPA and LGA showed better performance for older participants presenting cardiovascular risk factors. Results suggest that incorporating comprehensive cohort factors like diverse age, sex and participants with and without hypertension in the TD could enhance WML-based analyses and mitigate potential sources of bias. LPA and LGA are a fast and valid option for older participants with cardiovascular risk factors.

**Supplementary Information:**

The online version contains supplementary material available at 10.1007/s11357-024-01306-w.

## Introduction

White matter lesions (WMLs) of presumed vascular origin, also known as white matter hyperintensities (WMHs) due to their hyperintense appearance on T2-weighted, fluid attenuated inversion recovery (FLAIR) and proton density-weighted images, are common findings on brain magnetic resonance imaging (MRI) scans of older adults [1]. They are an expression of small vessel disease (SVD) [2]. Their prevalence increases with age [3, 4] and they are associated with cognitive impairment [5] and risk of dementia [6].

The assessment of WML for a clinical diagnosis, such as mild cognitive impairment or dementia [7], is typically performed by visual inspections of the MR scans using rating scales (e.g.: [8–11]). However, this method requires trained personnel, it is time-consuming, it has high intra- and inter-operator variability, and it provides semi-quantitative information [12–14]. Volumetric measurements of WML offer an improved alternative [14, 15]. While manual segmentation of WML is feasible for small studies, automated segmentation algorithms are necessary for large cohort datasets, such as the UK Biobank [16] or the German National Cohort (NAKO) [17]. In recent years, numerous algorithms have been developed for automatic WML segmentation [18]. An example is the well-established and widely used Brain Intensity AbNormality Classification Algorithm (BIANCA), a fully automated, supervised machine learning method based on the k-nearest neighbour (k-NN) algorithm, which mandates training datasets [19]. BIANCA allows to be trained by customised training datasets to then be applied to unseen brain images. Together with the training dataset, an initial set of parameters is needed. These parameters determine in which way the spatial and intensity information derived from the training data are utilised, as well as the number of training data points employed. Another example is the lesion growth algorithm (LGA), originally developed for segmenting lesions in multiple sclerosis (MS) patients. This is an unsupervised algorithm that has also been applied to segment the WML of cognitively unimpaired elderly individuals [20] and individuals with other conditions such as diabetes mellitus [21]. LGA employs T1 and FLAIR data to create a lesion belief map. After an initial user-determined parameter, the initial threshold kappa, is applied to the lesion belief maps, intensity outliers are identified. These outliers will then be expanded by analysing the intensity of neighbouring voxels. This is done by applying an iterated growth algorithm that uses maximum likelihood estimators to create a probability lesion map [22]. The algorithm stops when the estimator reaches a predefined limit. Therefore, LGA does not mandate training data, but it needs an initial user-selected parameter [22]. Another common algorithm used in the study of WML is the lesion prediction algorithm (LPA), a binary classifier using logistic regression, trained on data of 53 MS patients with severe lesion patterns [23]. LPA does not need initial parameters to be set nor training data, as the algorithm is already trained on a specific MS training dataset. Over the past years, many studies have focused on identifying the best automatic segmentation methodology, i.e., the one with the highest performance, whether supervised, unsupervised, automatic or semi-automated [18, 19, 22, 24–26]. Despite efforts to optimize these methods, an outstanding automatic method has not been identified yet [18, 27]. Although most of the algorithms have been tested individually [18], a comprehensive comparison across various WML segmentation algorithms is still needed to identify which ones accurately delineate WML patterns.

The occurrence of WML, however, has been shown to be significantly influenced by high blood pressure levels, hypertension, obesity, blood glucose levels, diabetes, age, sex, smoking and low physical activity [28–31]. Participants with levels of these factors associated with a less health conscious lifestyle tend to present a higher total WML volume. Automatic segmentation methods like BIANCA have been tested using random training datasets, e.g. [32] or datasets focused on the WML load, comprising participants with low, medium and high amount of WML [27, 33]. Yet, the information contained in training datasets might be highly related to the information that can be identified and ‘learned’ by the algorithm. If the training data is very specific, it might limit the algorithm’s knowledge and generalizability to other datasets, potentially introducing bias on the WML volume estimations. For example, if the selection is too narrow, such as including only participants with high WML load, the algorithm may be biased towards accurately segmenting only high amounts of WML and perform poorly for participants with low amount of WML. The algorithm could also tend to overestimate scans with a low WML amount. Similarly, if the training data consist solely of hypertensive participants, the algorithm performance may be low when applied to healthy individuals. Hence, it might be expected that the output of the algorithm is influenced by the training data, while in turn it is still unclear how the algorithm reacts to data, which it has not seen. Since groups of people with different levels of the above-mentioned lifestyle and risk factors likely present different patterns of WML, the composition of the training data might be crucial for the algorithms output. Thus, analysing differences in WML estimations using distinctly selected training datasets that represent various relevant factors influencing the WML load is relevant to evaluate the accuracy of the algorithms, yet it remains to be explored. Additionally, automated methods that do not require training datasets, such as LGA and LPA, have undergone testing and validation across various cohorts composed of cognitive impaired participants, individuals with dementia, and in healthy older adults (50–78 years) [20, 26, 27, 34, 35]. However, evaluating their performance on different subsamples within a cohort, composed of distinct relevant influencing factors, remains to be elucidated. This is crucial for determining whether the accuracy and performance of a particular algorithm remain consistent across the entire cohort or if it performs better only for participants presenting specific factors. Thus, determining which WML patterns—influenced by specific risk factors—can be reliably detected by which of the algorithms still needs to be elucidated.

Hence, the first aim of this study is to determine if the composition of the *training* data influences WML estimations. Specifically, we aim to discover if bias is introduced when the algorithm is trained on individuals with specific relevant factors. Since LGA and LPA do not require training, whereas BIANCA does, we will use BIANCA to address this question. We will *test* BIANCA when trained on different datasets, each one with a specific focus on a relevant influencing factor for WML load. Therefore, we will train BIANCA multiple times, each time on a group of individuals with a specific selection of relevant characteristics, i.e. age, sex, blood glucose levels, diabetes, blood pressure levels and hypertension. Here, we will also identify which composition of training data yields the highest performance.

The second aim is to evaluate the output and performance of three different algorithms, with respect to the presence of specific relevant characteristics in the *test* data; i.e. we aim to identify, if the different algorithms produce (in)accurate delineation when applied to specific subgroups of individuals. If so, we examine which characteristics in the *test* data are of concern for which of the algorithms. Therefore, we will compare the output and performance of BIANCA, LPA and LGA against each other when applied to individuals exhibiting specific influencing factors, i.e. age, sex, blood glucose levels, diabetes, blood pressure levels and hypertension. For this aim, BIANCA will be used with the training setup that yielded the highest performance in the 1st aim.

Results show that the composition of *training* datasets influences BIANCA’s WML estimations, introducing bias when the training data is very specific; e.g. when only trained on younger participants, WML load will systematically be underestimated. BIANCA’s highest performance was identified when the algorithm was trained on a group of individuals presenting all different relevant influencing factors. LPA and LGA performed poorly when applied to participants younger than 67 years of age (mean DSI < 0.4) but their performance improved for older participants with cardiovascular risk factors. BIANCA outperformed (mean DSI > 0.7) LPA and LGA when applied to different groups of individuals presenting all different cohort characteristics.

## Materials and methods

For this study, we used three established and available segmentation algorithms, i.e. BIANCA, LPA and LGA, since they are freely available, are widely used in the community [19, 21, 27, 32, 34, 36–43] and use different methodological approaches for segmenting the WML.

### Participants

The data used in this study is sourced from the population-based 1000BRAINS cohort [44], which focuses on investigating structural and functional variations in the normal aging brain. Participants were recruited from the Heinz-Nixdorf-Recall (HNR) study and the related HNR-Multi-Generation-study [45]. This dataset includes a wide range of epidemiological information, such as neuropsychological tests, life quality, mood and daily activities, as well as laboratory, genetic, clinical, socioeconomic and environmental data.

Inclusion criteria for the present study required participants to be free from strokes and provide complete data, including FLAIR and T1 images, along with age, sex, blood glucose levels, systolic and diastolic blood pressure levels, diabetes diagnosis and hypertension diagnosis. Initially, there were 1314 participants, but 55 were excluded due to incomplete laboratory information (see ‘Influencing factors’ section), 65 lacked the complete set of T1 + FLAIR modalities and 28 were excluded because of any type of tissue defects post stroke. In total, 1166 participants (mean age = 60, range 18–87, female (F):male (M) 523:643) from the first visit, as detailed in [44], were included in this study. Participants belonging to the same family were addressed in the statistical analysis described in a later section. All participants provided informed consent in accordance with the Declaration of Helsinki. The study protocol of 1000BRAINS was approved by the ethics committee of the University of Duisburg-Essen.

### MRI data

MRI brain scans were conducted using a 3-T MR scanner (Siemens Tim-TRIO; for the whole protocol, see Caspers et al. 2014). The sequences included in this study were as follows: a 3D T1-weighted MPRAGE (176 slices, TR = 2.25 s, TE = 3.03 ms, TI = 900 ms, FoV = 256 × 256 mm^2^, flip angle = 9°, voxel resolution = 1 × 1 × 1 mm^3^) and a clinical T2-weighted FLAIR (25 slices, TR = 9 s, TE = 100 ms, FoV = 220 × 220 mm^2^, flip angle = 150°, voxel resolution = 0.9 × 0.9 × 4 mm^3^).

### Relevant influencing factors

Given the associations between WML and age, sex, high blood glucose levels, diabetes, high blood pressure and hypertension demonstrated in previous studies [29, 31, 46–48], we included these influencing factors in the present study. The following information was considered for each participant: age, sex, systolic blood pressure (mmHg), diastolic blood pressure (mmHg), whether participants had received a diagnosis of hypertension, the participants’ respective medication against hypertension (if applicable), and blood glucose levels (mg/dL) and whether subjects had a diagnosis of diabetes mellitus. A participant was considered to have diabetes if having a respective confirmed diagnosis. Subjects with confirmed diagnosis of hypertension or taking medication against hypertension were considered to have hypertension.

### Evaluating the impact of relevant influencing factors on automatic WML estimations

#### Aim 1: influence of training data

To determine if the composition of the *training* data influences BIANCA’s WML estimations, we created 15 *training* datasets, each showcasing a unique characteristic that could potentially influence BIANCAs’ outcome. We then trained the algorithm 15 times, each time for each training dataset, and compared the resulting WML estimations of the whole 1000BRAINS cohort. First, we selected a subsample characterised solely by participants’ age, ensuring that no other relevant factor was present. Specifically, we excluded participants with hypertension, systolic blood pressure exceeding 140 mmHg [49], diabetes or a blood glucose level above 126 mg/dL [50]. We grouped these participants into six training datasets: age 18 (18–37 years), age 37 (37–47 years), age 47 (47–57 years), age 57 (57–67 year), age 67 (67–77 years) and age 77 (77–87 years) (as shown in Table 1). We followed the same approach for studying the influence of all other relevant factors, i.e. selecting one factor of interest and keeping all other factors stable in the respective subsample. For exploring the influence of sex, we selected two subsamples of participants above 60 years old [51]: one without cardiovascular factors (hypertensions and diabetes) and another one with these factors. We then grouped the participants according to their sex, resulting in four more training datasets: ‘males with no cardiovascular factors’ (male–no CF), i.e. males with no hypertension and/or diabetes, with blood glucose level below 126 mg/dL, and with systolic blood pressure below 140 mmHg, and ‘females without cardiovascular factors’ (female–no CF), i.e. we created two ‘healthy’ older training datasets, one of males and another one of females; and, on the other hand, ‘males with cardiovascular factors’ (male–CF), i.e. with hypertension or diabetes or with blood glucose level above 126 mg/dL or with systolic blood pressure above 140 mmHg, and ‘females with cardiovascular factors’ (female–CF), i.e. two older groups, one with males and another one only with females, both presenting relevant cardiovascular factors (see Table 1). For exploring the influence of particular cardiovascular factors on older participants, we created four training datasets with individuals above 60 years old [51]. One comprising participants with a confirmed diabetes diagnosis or with blood glucose level exceeding 126 mg/dL [31, 50], we called this training dataset ‘diabetes’; another one comprising participants diagnosed with hypertension or systolic blood pressure above 140 mmHg [49], we called this dataset ‘hypertension’, and two ‘control’ subgroups, ‘control diabetes’ and ‘control hypertension’, to compare against the ‘diabetes’ and ‘hypertension’ training datasets. The ‘control’ datasets consisted of ‘healthy’ participants with blood glucose level below 126 mg/dL and systolic blood pressure below 140 mmHg. Finally, we created one last training dataset comprising participants with a uniform age distribution raging from 18 to 87, mixed sex, with and without the presence of cardiovascular factors (hypertension and diabetes), we called this training data set ‘TD120’ (as is constituted by 120 participants); see Table 1 for a summary of the training datasets. Lastly, the number of participants in each training dataset was selected in order to maintain the same prevalence of these factors in the 10000BRAINS cohort.

**Table 1:** Description of the *training* and validation datasets

Training and validation datasets	No. of participants	Glucose levels	Diabetes	Systolic blood pressure	Hypertension
Age					
Age 18 (18–37)	10	< 126 mg/dL	0	< 140 mmHg	0
Age 37 (37–47)	10	< 126 mg/dL	0	< 140 mmHg	0
Age 47 (47–57)	13	< 126 mg/dL	0	< 140 mmHg	0
Age 57 (57–67)	20	< 126 mg/dL	0	< 140 mmHg	0
Age 67 (67–87)	11	< 126 mg/dL	0	< 140 mmHg	0
Age 77 (77–87)	8	< 126 mg/dL	0	< 140 mmHg	0
Sex—age > 60 years old					
Female–CF	20	> 126 mg/dL	1	> 140 mmHg	1
Male–CF	25	> 126 mg/dL	1	> 140 mmHg	1
Female–no CF	11	< 126 mg/dL	0	< 140 mmHg	0
Male–no CF	14	< 126 mg/dL	0	< 140 mmHg	0
Cardiovascular risk factors—age > 60 years old					
Diabetes	14	> 126 mg/dL	1	< 140 mmHg	0
Control Diabetes	25	< 126 mg/dL	0	< 140 mmHg	0
Hypertension	27	< 126 mg/dL	0	> 140 mmHg	1
Control HYPERTENSION	31	< 126 mg/dL	0	< 140 mmHg	0
TD120	120	All levels	0 and 1	All levels	0 and 1

Female–CF: females with cardiovascular factors; male–CF: males with cardiovascular factors; female–no CF: females with no cardiovascular factors; male–no CF: males with no cardiovascular factors

#### Aim 2: impact of relevant factors on test data

To identify if the presence of specific factors in the *test* data leads to (in)accurate delineation, we created 13 *test* datasets, each displaying a unique characteristic. We then compare the WML estimations and performance of BIANCA, LPA and LGA within each group of individuals. Similar to the creation of the training datasets for aim 1, we first selected a subsample characterized solely by participants’ age, ensuring that no other relevant factors were present. We grouped these participants into five test datasets: age 18 (18–37 years), age 37 (37–47 years), age 47 (47–57 years), age 57 (57–67 year) and age 67 (67–87 years) (as shown in Table 2). Secondly, we grouped participants above 60 years old [51] into four test datasets based on their sex and presence of cardiovascular factors: ‘males with no cardiovascular factors’ (male–no CF), ‘females without cardiovascular factors’ (female–no CF), ‘males with cardiovascular factors’ (male–CF), i.e. with hypertension or diabetes or with blood glucose level above 126 mg/dL or with systolic blood pressure above 140 mmHg, and ‘females with cardiovascular factors’ (female–CF) (see Table 2). Lastly, for exploring specifically the impact of particular cardiovascular factors on older participants, we created four test datasets. The first one, comprising participants with a confirmed diabetes diagnosis or presenting a blood glucose level exceeding 126 mg/dL [50], was called ‘diabetes’. The second one, integrating participants diagnosed with hypertension or presenting a systolic blood pressure above 140 mmHg [49], was denominated with the name ‘hypertension’. And two ‘control’ test datasets were called ‘control diabetes’ and ‘control hypertension’. These ‘control’ test datasets consisted of ‘healthy’ participants with blood glucose level below 126 mg/dL and systolic blood pressure below 140 mmHg. All participants in these test datasets were above 60 years old [51] (see Table 2 for a summary of the test datasets).

**Table 2:** Description of the 13 *test* datasets

Test datasets	No. participants	Glucose levels	Diabetes	Systolic blood pressure	Hypertension
Age					
Age 18 (18–37)	86	< 126 mg/dL	0	< 140 mmHg	0
Age 37 (37–47)	78	< 126 mg/dL	0	< 140 mmHg	0
Age 47 (47–57)	113	< 126 mg/dL	0	< 140 mmHg	0
Age 57 (57–67)	175	< 126 mg/dL	0	< 140 mmHg	0
Age 67 (67–87)	95	< 126 mg/dL	0	< 140 mmHg	0
Sex–age > 60 years old					
Female–CF	180	> 126 mg/dL	1	> 140 mmHg	1
Male–CF	286	> 126 mg/dL	1	> 140 mmHg	1
Female–no CF	121	< 126 mg/dL	0	< 140 mmHg	0
Male–no CF	101	< 126 mg/dL	0	< 140 mmHg	0
Cardiovascular risk factors—age > 60 years old					
Diabetes	52	> 126 mg/dL	1	< 140 mmHg	0
Control diabetes	222	< 126 mg/dL	0	< 140 mmHg	0
Hypertension	167	< 126 mg/dL	0	> 140 mmHg	1
Control hypertension	222	< 126 mg/dL	0	< 140 mmHg	0

1 indicates a positive diagnosis and 0 no diagnosis. Female–CF: females with cardiovascular factors; male–CF: males with cardiovascular factors; female–no CF: females with no cardiovascular factors; male–no CF: males with no cardiovascular factors

### Additional step: repeating aim 1 on characterised subsamples

As an additional step, after creating the characterised subsamples in aim 2, we repeated our analyses for aim 1 on each distinctive test dataset. This means we repeated analyses for aim 1 13 times, but instead of applying it to the entire 1000BRAINS cohort, we applied it to each characterised *test* dataset. This approach allowed us to analyse whether the influence of the relevant factors in the *training* data differed when the characteristics of the *test* subsamples changes.

### Manual WML segmentation

**Fig. 1:**
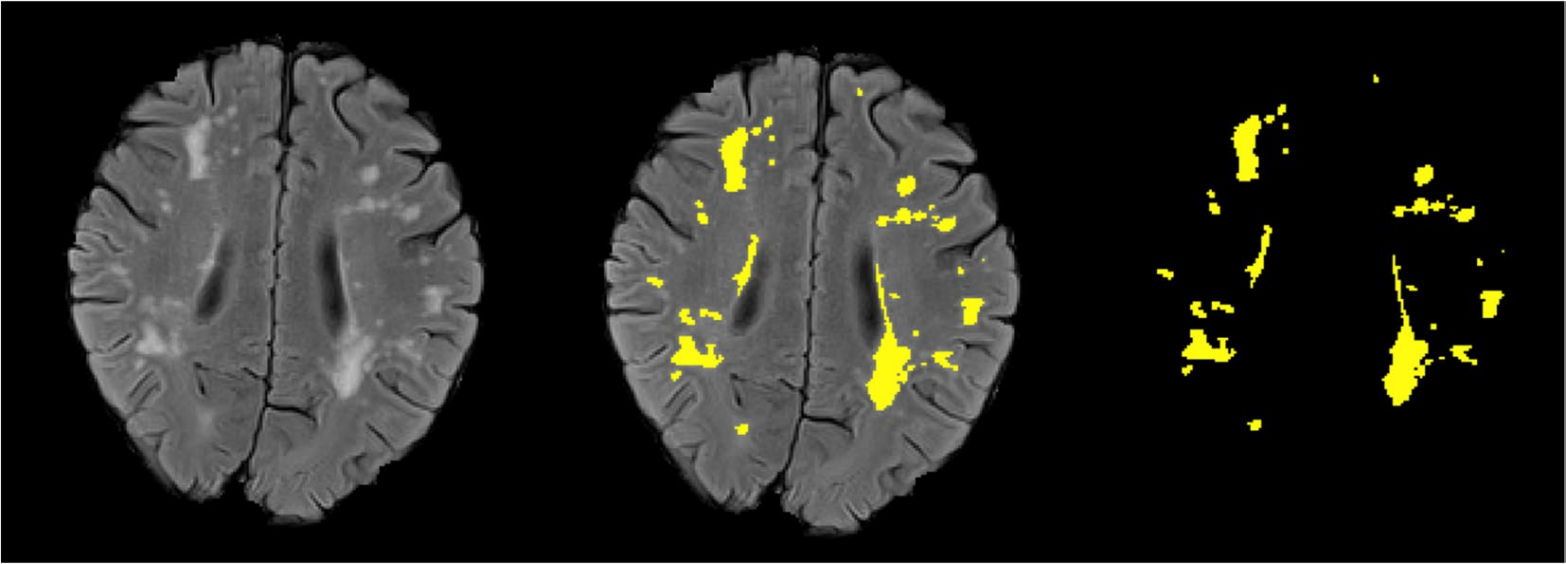
Example of a manual segmentation on a FLAIR image

To examine whether BIANCA’s WML estimations are influenced by the presence of relevant factors in the training data, we manually segmented the WML on FLAIR modality of the participants conforming each training dataset shown in Table 1 (total of 120 participants). We did this with FSLeyes, a tool from FSL (https://fsl.fmrib.ox.ac.uk/fsl/fslwiki). Binary masks were generated with a value of 0 for non-WML voxels and 1 for WML voxels. Examples of manually segmented masks are shown in Fig. 1.

To analyze the performance of BIANCA, LPA and LGA on distinct characterised subsamples, we examined the degree of overlap between WML masks provided by the algorithms and manually segmented WML masks. For this purpose, we employed the same data created in the previous step as validation data, i.e. the 120 manually segmented scans in FLAIR space. Since LGA uses T1 modality as base reference, we also required validation data in T1 space. Therefore, we co-registered the manually segmented WML masks in FLAIR space to T1 space using FLIRT from FSL for modality co-registration (FLAIR to T1). Detailed information on the number of participants in each validation dataset is provided in Table 1.

### Automated WML segmentation algorithms

To address the first aim, we opted for one of the most widely used and established algorithms in the literature [27, 32, 39, 40, 52, 53], BIANCA [19]. BIANCA requires data pre-processing, selection of initial parameters and to be trained.

The pre-processing steps involved tools from FSL (http://fsl.fmrib.ox.ac.uk/fsl) [54]. We utilised BET to produce brain extracted images in FLAIR and T1 modality, FLIRT for modality co-registration (T1 to FLAIR) using linear rigid-body registration (6 degrees), and normalization to the MNI152 standard template [55].

Regarding the initial parameters, we used T1 and FLAIR modalities, with FLAIR as the reference base modality. We followed the options recommended by [19] to optimise the dice similarity index (DSI) and false positive ratio. This included setting spatial weighting (sw) to 1 (default), no patch and selecting no border (excluding three voxels close to the lesion’s edge) for the location of non-lesion training points. We used a fixed and unbalanced (FU) number of training points, with 5000 for the number of lesion points and 25,000 for non-lesion points per training subject. The total WML volume, and hence the BIANCA estimation, was obtained by applying a threshold of 0.9 [19] to the lesion probability map, which constitutes the output of the algorithm. Further details can be found in [19].

BIANCA is trained by creating a feature space that includes both intensity and spatial features from the lesion and non-lesion voxels determined in the training data. Feature vectors for both classes, WML and non-WML, are created for each of the selected number of training points. Once the ‘training’ vectors are established in the feature space, classification of unseen voxels (unseen images) is performed by creating its own feature vector and measuring the distance to the 40 nearest training feature vectors (k-nearest neighbour). Therefore, by using each specific characterised training data (shown in Table 1), BIANCA generated specific training feature vectors linked to each relevant factor. This approach allowed us to assess WML estimation differences when training data characteristics changed.

To address the second aim, we selected algorithms that, like BIANCA, are well established and broadly used in the community [19, 21, 27, 32, 34, 36–43]. These algorithms include BIANCA itself, the Lesion Prediction Algorithm (LPA) [23] and the Lesion Growth Algorithm (LGA) [22].

In this case, BIANCA was trained on participants with a uniform age distribution ranging from 18 to 87, mixed sex, with and without the presence of cardiovascular factors, since this characterised dataset yelled the highest performance in aim 1.

LPA only requires the input modality: FLAIR. It also presents the option to include another modality as the base reference image. In this study, we tested the algorithm with two options, using only FLAIR modality and a combination of FLAIR + T1, using T1 as base modality. We applied a threshold of 0.5 [23] to the lesion probability maps.

LGA requires T1 modality as a reference image along with FLAIR images. An initial threshold (kappa), user-determined, is needed. In our study, we selected a kappa value of 0.25 [22] and applied a threshold of 0.3 [22] to the lesion probability maps [22, 23].

We applied BIANCA, LPA and LGA to the 13 characterised test datasets (described in Table 2) then compare their outputs and performance within each characteristic.

### Statistical analysis

For aim 1, to determine whether BIANCA estimations are influenced by the presence of relevant factors in the *training* data, we compared the estimated WML volumes obtained with each training dataset when the algorithms was applied to the 1000BRAINS cohort and to each characterised subsample depicting different age distribution, stratified by sex, with and without the presence of cardiovascular factors (as shown in Table 2). Specifically, we examined how different characteristics present in the *training* data influenced the results obtained by BIANCA. We conducted these comparisons using mixed ANOVAs with Bonferroni post hoc tests. We applied this test to address the participants who are related to each other (between-subjects factor) and the variance introduced by the different training datasets (within-subjects factor).

We also analysed the performance in each case, to identify which composition of training data yielded the highest performance. We measured the degree of overlap between WML masks provided by BIANCA and WML manually segmented masks (see validation datasets in Table 1). We employed a specific metric for this purpose, the dice similarity index (DSI). The DSI is calculated as twice the number of voxels in the intersection of manual and algorithm masks divided by the sum of voxels manually segmented and algorithm segmented voxels. This choice aligns with previous studies, which have identified the DSI as the most robust indicator of overlap between the manual mask and the estimated mask [19].

For aim 2, to identify if the presence of specific factors in the *test* data leads to (in)accurate delineation, we analysed the output differences of BIANCA, LPA and LG, as well as their performance when applied to individuals exhibiting different age distribution, stratified by sex, with and without the presence of cardiovascular factors (details of individuals characteristics are shown in Table 2).

Regarding the output differences, we compared the outcomes of BIANCA versus LPA when using FLAIR modality only, versus LPA when using both T1 and FLAIR modalities, and versus LGA, within each characterised *test* data described in Table 2. For instance, we considered the test subgroup ‘hypertension’ as explanatory factor, and WML volume estimations as dependent variable (within-subjects). We analysed these differences using mixed between-within participants ANOVAs with Bonferroni post hoc tests.

Regarding the performance, we measured the degree of overlap between WML masks provided by the different algorithms and manually segmented WML masks (see validation datasets in Table 1) employing the DSI.

## Results

### Aim 1: influence of training data

**Fig. 2:**
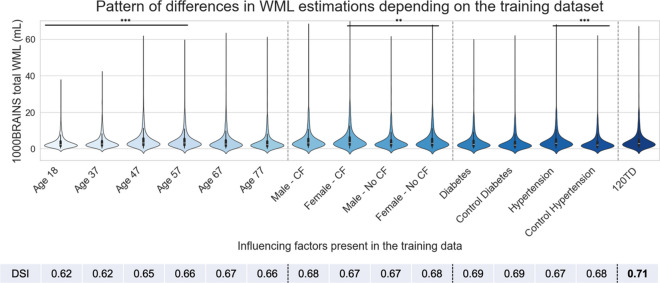
Training data characteristics influences BIANCA’s WML estimations. Violin plots present the distribution of the total estimated WML volume as median values and quartiles when BIANCA is trained on 15 different training data sets (*x*-axis) and applied to the whole 1000BRAINS cohort. **0.001 < *p* ≤ 0.01; ***: 0.0001 < *p* ≤ 0.001. Violin plots were made with seaborn library

First, we focused on the question on how different characteristics of *training* datasets would influence the WML estimations within one such algorithm, BIANCA. When exploring BIANCA’s output differences a specific pattern of significant differences emerged for all test datasets, we found that different training datasets based on different age distribution (*p* < 0.001), stratified by sex (*p* < 0.01), and with and without the presence of hypertensive individuals (*p* < 0.001) yielded different results as shown in Fig. 2.

Notably, WML volumes exhibited significant increases when BIANCA was trained on older participants, i.e., specifically on participants between 47 and 67 years old compared to participants between 18 and 47 years old (*p* < 0.001), see Fig. 2. Additionally, higher WML volumes were also observed when the algorithm was trained on females with cardiovascular risk factors, compared to training on males and females without cardiovascular factors (*p* < 0.01). Furthermore, higher WML volumes were observed when the algorithm was trained on the ‘hypertension’ training dataset in comparison to when trained on ‘healthy’ participants (‘control hypertension’ training dataset (*p* < 0.001)).

When identifying peak performances, we spotted that the highest similarities between output mask and manual segmentation (DSI ≥ 0.7) was achieved when the *training* dataset had a uniform age distribution ranging from 18 to 87, mixed sex, individuals with and without the presence of cardiovascular factors, i.e. when BIANCA was trained on ‘TD120’ (see Fig. 2).

### Aim 2: impact of relevant factors on test data

Secondly, we focused on the question how different characteristics of *test* datasets would impact on WML estimations of three algorithms BIANCA, LPA and LGA. We examined the output and performance of the algorithms in each characterised *test* dataset described in Table 2.

Therefore, we first compared the estimated WML volumes between BIANCA vs. LPA using only FLAIR vs. LPA using T1 and FLAIR vs. LGA.

#### BIANCA vs. LPA vs. LGA—impact of ‘age’ on test data

**Fig. 3:**
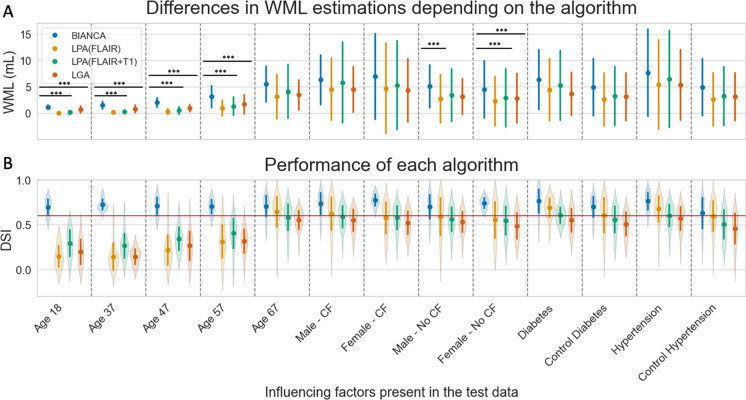
Impact of test data characteristics on outputs and performance of BIANCA, LPA and LGA. Presented are **A** mean WML estimations (ml) for each test datasets (*x*-axis) depending on the four algorithms with standard deviations. **B** Performance of each algorithm is measured by the DSI indicating the similarity between the algorithms WML segmentation mask and the manual segmentation mask, where violin plots illustrate the distribution of DSI with mean values and standard deviations. Abbreviations: LPA(FLAIR) = LPA using only the FLAIR modality, LPA (FLAIR + T1) = LPA using the FLAIR and T1 modalities; ***0.0001 < *p* ≤ 0.001. Violin plots and point plots were made with seaborn library

We found a significant difference (*p* < 0.001) between the output of BIANCA and that of LPA for participants under 67 years of age, i.e. for test datasets ‘age18’, ‘age37’, ‘age47’ and ‘age57’ (see Fig. 3A). Specifically, LPA consistently provided lower WML estimations when compared to BIANCA’s output which was evident in the WML distributions in Fig. 3A. When comparing output masks with the manual segmentations, this discrepancy was also reflected in high similarities for BIANCA (mean DSI > 0.7) compared to lower similarities for LPA with only FLAIR modality (mean DSI < 0.4), and to LPA with FLAIR + T1 modality (mean DSI < 0.4); see Fig. 3B. Furthermore, we observed that LGA’s output showed significantly (*p* < 0.001) lower WML estimations than BIANCA’s for participants under 67 years of age (Fig. 3A). This is consistent with low similarities observed for LGA when applied to participants under 67 years of age (mean DSI < 0.3, Fig. 3B). BIANCA, however, showed highest similarities (mean DSI > 0.7) when applied to all age distributions (Fig. 3B).

#### BIANCA vs. LPA vs. LGA—impact of ‘sex’ on test data

Results show significant differences (*p* < 0.001) between the outputs of BIANCA and LPA when using only the FLAIR modality. Specifically, in older males without cardiovascular factors (see Fig. 3A), BIANCA yielded higher WML estimation volumes compared to LPA. At the same time, higher DSI (> 0.7), i.e. a higher similarity, is observed for BIANCA’s output compared to LPA’s (FLAIR) output (DSI < 0.6, Fig. 3B). Furthermore, significant differences (*p* < 0.001) between the outputs of BIANCA, LPA and LGA were observed for older females without cardiovascular factors: notably, BIANCA shows higher WML volumes compared to all other algorithms (see Fig. 3A). Again, this is in line with higher observed performance for BIANCA (mean DSI > 0.7) compared to all other algorithms (mean DSI < 0.6, see Fig. 3B). No significant differences were observed between LGA and LPA’s output when they were applied to older males and females with and without cardiovascular factors (Fig. 3A). For all sex-stratified test data, BIANCA showed the highest performance (mean DSI > 0.7, see Fig. 3B).

#### BIANCA vs. LPA vs. LGA—impact of ‘cardiovascular factors’ on test data

In contrast to our previous findings, we did not observe any significant difference between the algorithms’ output when they are applied to older individuals with high blood glucose levels, diabetes, high blood pressure levels, hypertension and healthy controls, i.e. for test datasets ‘diabetes’, ‘control diabetes’, ‘hypertension’ and ‘control hypertension’ (see Fig. 3A). LPA(FLAIR), LPA(FLAIR + T1) and LGA showed a good performance (mean DSI, 0.55–0.69) for older individuals with high blood glucose levels, diabetes, high blood pressure levels or hypertension, and BIANCA outperformed all of these with a mean DSI > 0.7; see Fig. 3B.

### Additional step: repeating aim 1 on characterised subsamples

When repeating the analysis of aim 1 on the subsamples created on aim 2, the same pattern of differences found in aim 1 was found in almost all *test* datasets. There was a slight difference in the pattern found in participants between 57 and 87 years old. Here, higher WML volumes were observed when the algorithm was trained on females with cardiovascular risk factors, compared to training only on males without cardiovascular factors (*p* < 0.01). Please refer to the supplement material (Fig. s1E and D) to see this slight variation in the pattern of WML estimated differences. When looking at each subgroup individually (Fig. s1–s3), we noticed that the highest similarities between output mask and manual segmentation (DSI ≥ 0.7) was achieved when the *training* dataset had a uniform age distribution ranging from 18 to 87, mixed sex, individuals with and without the presence of cardiovascular factors, i.e. when BIANCA was trained on ‘TD120’, replicating the same result found in aim 1 (Fig. s1–s3). We also observed that high similarities were achieved when *test* datasets presented similarity with the *training* datasets, e.g. regarding age or (non-)presence of risk factors. This was most observable in the age 37 *test* subgroup, where WML estimations where most accurate when using *training* data from participants under 37 years of age. For most other groups, the range was wider; e.g. for the youngest age decade (‘age 18’ subgroup), the most accurate WML estimations were achieved when using a *training* dataset comprising participants under 47 years old without cardiovascular risk factors. Please refer to the supplement material (Fig. s1–s3) for a detailed depiction of all DSI results.

## Discussion

In this study, we aimed to investigate the effect of different relevant influencing factors on automatic WML estimations in a large sample of normal aging participants. We selected three freely available and widely used algorithms, BIANCA, LPA and LGA, and compared their outputs and performance under different conditions.

The first aim of this study was to determine whether automatic WML estimations are influenced by the presence of relevant factors in the *training* dataset. Specifically, we found that (i) *training* datasets induce bias when they consist of a narrow selection of characteristics, i.e. including only older participants, females with cardiovascular factors or only hypertensive individuals, as shown in our analyses comparing 15 different training datasets within BIANCA. Trained on these datasets, BIANCA overestimated WML volumes compared to when it was trained on younger participants, opposite sex and control individuals. Moreover, BIANCA’s best performance was achieved when trained on 120 individuals from all ages, both sexes and including individuals without the presence of cardiovascular factors.

The second aim was to compare WML estimations and performance of BIANCA, LPA and LGA when applied to *test* subgroups with specific risk factor profiles. Here, (ii) WML estimations of LPA and LGA differ significantly from BIANCA; they underestimated the total WML volumes, e.g. in subjects under 67 years of age or older females without cardiovascular risk factors. (iii) LPA and LGA showed a poor performance when applied to subjects under 67 years of age without cardiovascular risk factors (DSI < 0.4). BIANCA showed a robust and the highest performance (DSI > 0.7) across all subgroups with specific risk factor profiles.

The necessity for a reliable automated segmentation is evident by the cumulus works dedicated to standardize the evaluation of WML load [18, 27, 56]. Despite the number of proposed methods [19, 22, 23, 25, 37, 57–69] and the attempts of improving them, an outstanding algorithm has not been recognized yet [56, 70]. Caligiuri et al. (2015) [18] compared 34 different WML automatic methods, including supervised learning algorithms, unsupervised algorithms and automated and semi-automated algorithms. They established that many of these algorithms are not freely available, and they have been validated mostly with small samples and are study and/or protocol specific. In our study, we choose BIANCA, LPA and LGA because they are freely available, they are commonly used in the community [21, 27, 32, 34, 36–43, 71], they are fully automated and the major difference regarding their use is that LPA and LGA do not require training data; meanwhile, BIANCA does. We aimed at providing a more differentiated perspective on the usage of such algorithms beyond the question of one optimal algorithm, but focusing on the relevance of potential sources of bias for algorithm performance and outcome when used in large population-based samples.

Regarding the first aim of the present study, we showed a discernible pattern of significant differences in BIANCA’s WML volume estimations emerged based on different *training* datasets related to age, sex, and hypertension. Notably, significantly higher WML loads were observed when BIANCA was trained on older individuals (Fig. 2), compared to when it was trained on younger individuals. This observation could indicate that the age composition of the *training* dataset plays an important role in the accuracy of WML volume estimations. Specifically, relying solely on older adults or exclusively on younger adults within a cohort could introduce bias, potentially leading to either an overestimation or underestimation of WML volumes. This emphasizes the importance of constructing a well-balanced training dataset that encompasses a diverse representation of ages to avoid bias WML estimations.

Moreover, elevated WML volumes were noted when the algorithm was trained on females with cardiovascular risk factors (Fig. 2). Similarly, higher WML estimations were observed when the training sample consisted of individuals with high blood pressure or hypertension. Previous studies indicate a higher WML load in participants with hypertension [29, 53]. This together with our results suggests that including only participants with these factors in the training data has the potential to markedly alter the estimations of WML volume estimations. These findings highlight the substantial influence of *training* data characteristics on WML volume extraction, emphasizing the need for a comprehensive training data where male and female participants with and without cardiovascular factors are needed in order to avoid bias in the WML volume estimations. Accentuating this point, the highest performance (DSI, 0.70–0.78) was observed when BIANCA was trained on a group of individuals with uniform age distribution ranging from 18 to 87, mixed sex, with and without the presence of cardiovascular factors, representing a balanced and proportioned characterization of the influencing factors present in the 1000BRAINS cohort.

Regarding the second aim, we could show that LPA and LGA underestimated the total WML volumes in participants under 67 years of age and in older females without cardiovascular risk factors. This aligns with the low performance (DSI < 0.4) of these algorithms on individuals under 67 years old. To evaluate performance as moderate or good, a DSI of > 0.6 is usually expected [27]. Heinen et al. (2019) [26] explored the performance of LGA and LPA in a study involving 60 subjects with vascular cognitive impairment. Their findings indicated a relatively lower performance for LGA when compared to LPA. Our findings from a large group of normal aging participants also indicate a lower performance for LGA compared to LPA, thus expanding previous evidence from clinical conditions to the normally aging population. This consistency across studies emphasizes the importance of considering the algorithm’s performance characteristics in specific demographic and clinical contexts.

BIANCA has been tested, validated and applied in many different cohorts [27, 32, 39, 53]. A recent study by Hotz et al. (2022) [32] explored WML estimation in 232 healthy subjects aged 64–87, employing three different algorithms, including BIANCA. They reported a mean DSI of 0.6 using a random training dataset from the cohort comprising 16 FLAIR images with manually segmented lesions. In our study, we tested 15 combinations of relevant factors in the training dataset, identifying the highest performance (mean DSI > 0.7) when the training data characteristics were similar to those of the cohort.

LPA has been trained on 53 multiple sclerosis patients with severe lesion patterns, with a total WML volumes higher than 10 mL, reflecting a comparably high amount of WML load. Similar to LPA, LGA was originally developed for lesion segmentation in patients with multiple sclerosis [22]. However, it has also been used for WML segmentation in, e.g. cognitively unimpaired older adults [38], individuals with dementia and cognitive impairment [27] and individuals with diabetes [21]. It has been established that methods trained on multiple sclerosis patients perform relatively well when applied to geriatric patients [57]. This aligns to the results we observed, where LPA’s performance improves for participants > 60 years of age with cardiovascular risk factors (who also present high WML burden). Yet, the performance of the algorithm is not optimal for participants under 67 years of age without cardiovascular risk factors (DSC < 0.4). These changes in LPA’s performance are comparable with the drop in performance for LGA. With LPA being pre-trained on multiple sclerosis patients with high amounts of WML, accurate delineations of WML in normally aging participants might be limited, as boundaries of multiple sclerosis lesions present with more clearly delineable edges as compared to age-related WML [18].

Our study thus would allow to conclude that LPA and LGA are suboptimal for automatic segmentation of WML for participants under 67 years of age without cardiovascular risk factors. However, they can be a very good and fast choice to estimate WML on participants > 60 years of age with cardiovascular risk factors, particularly LPA since it presented higher performance than LGA and uses the pretrained algorithm as implemented in the lesion segmentation toolbox (LST).

### Limitations

While we compared three widely used algorithms (BIANCA, LPA and LGA), there are other segmentation methods available in the literature. Our study focused on these three algorithms due to their common usage and availability. Different algorithms may yield different results, and future research could explore additional methods. The performance of machine learning algorithms, like BIANCA, can be highly dependent on the quality and representativeness of the training dataset. While we attempted to create a diverse training dataset based on the influencing factors, there may still be factors not considered in our study that could affect segmentation accuracy. In this study, the manual delineations were performed by a trained physicist specialized in medical image analysis supervised by a physician who selectively checked delineations; i.e. there was only one independent rater; therefore, there were no consensus procedure and no interrater reliability measure. The findings of this study are based on a specific cohort of normal aging individuals and may not fully generalize to other populations, such as clinical patients or individuals with neurodegenerative diseases. The impact of influencing factors on WML estimation may differ in different populations. Lastly, the data used is cross-sectional, and it may be of high interest to study the intra-subject variability of WML segmentation in relation to these influencing factors in a longitudinal dataset.

## Conclusion

Our study provides insights to minimizing sources of bias that may influence the white matter lesion estimations when using three freely available segmentation algorithms: LGA, LPA and BIANCA.

Based on our results, we see the importance of considering a comprehensive characterization of the sample dataset with special importance in lifestyle and influencing factors to the segmentation methodology to avoid sources of bias in the estimations of WMLs. Specifically, we encourage the users of BIANCA to prepare training datasets that embed, in a smaller scale, a representation of the distribution in terms of age, sex and hypertension. LGA showed the lowest performance compared to LPA and BIANCA. LPA proved to be a suitable and fast alternative for participants with certain characteristics, e.g. above 60 years of age with cardiovascular risk factors. BIANCA presented the highest performance across all the subgroups compared to LPA and LGA. However, it is important to note that BIANCA’s preparation of the training dataset takes time and expertise.

We suggest to be aware of the changes in WML estimations and accuracy when applying the LPA or LGA to subjects under 60 years of age.

In the future, it would be of interest to investigate how other influencing factors such as obesity, cholesterol levels, physical activity, smoking or genetics, e.g. APOE status, could impact automatic WML estimations.

## Supplementary Information

Below is the link to the electronic supplementary material.

Supplementary file1 (DOCX 5571 kb)

## Data Availability

The datasets generated and/or analyzed during the current study are available from the Heinz-Nixdorf-recall (HNR) and the related HNR-Multi-Generation-study committee to other scientists on request in anonymized format and according to data protection policy in the ethics agreement.
