# Supplementary material for: Identifying sources of bias when testing three available algorithms for quantifying white matter lesions: BIANCA, LPA and LGA
Source: GeroScience. 2024 Aug 8;47(1):1221–37. doi: 10.1007/s11357-024-01306-w (PMC11872996; doi:10.1007/s11357-024-01306-w)
Supplement: Supplementary file 1 — Supplementary file1 (DOCX 5571 kb) [file 11357_2024_1306_MOESM1_ESM.docx]

**Supplementary material**

**Identifying sources of bias when testing three available algorithms for quantifying White Matter Lesions: BIANCA, LPA & LGA**

1. **Results**

Additional step: repeating aim 1 on characterised subsamples


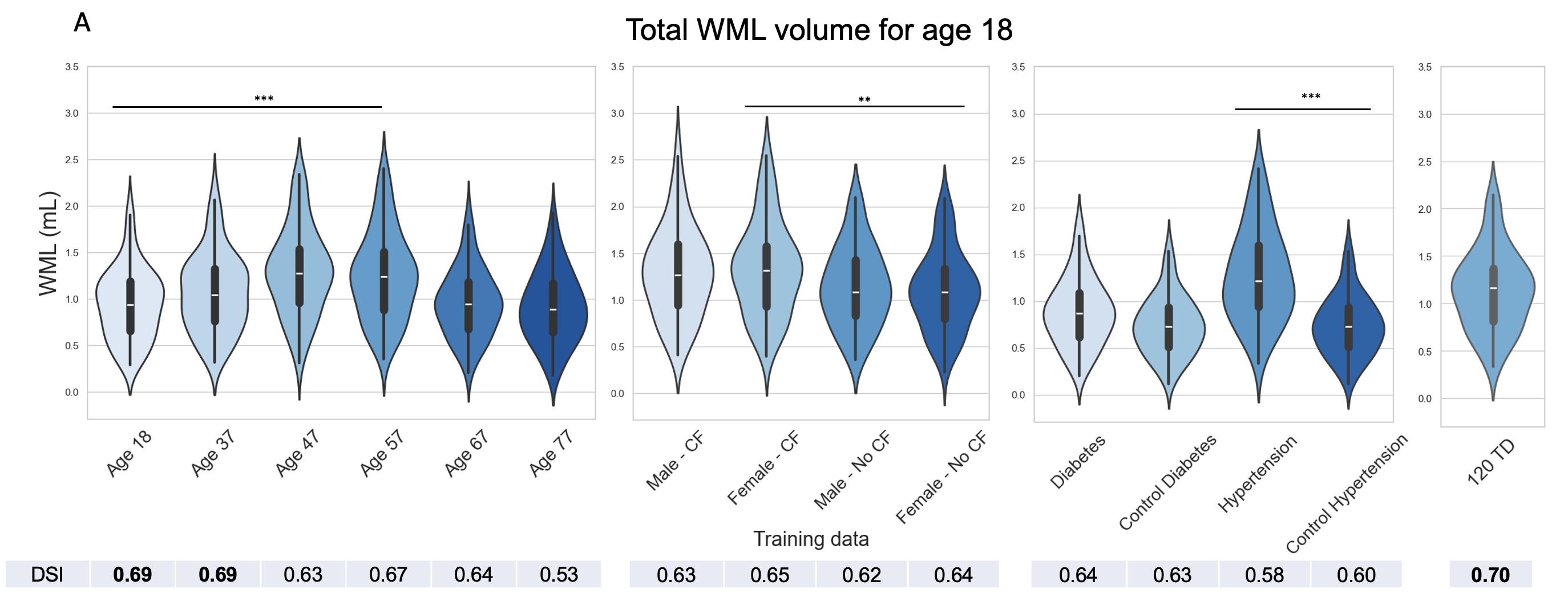


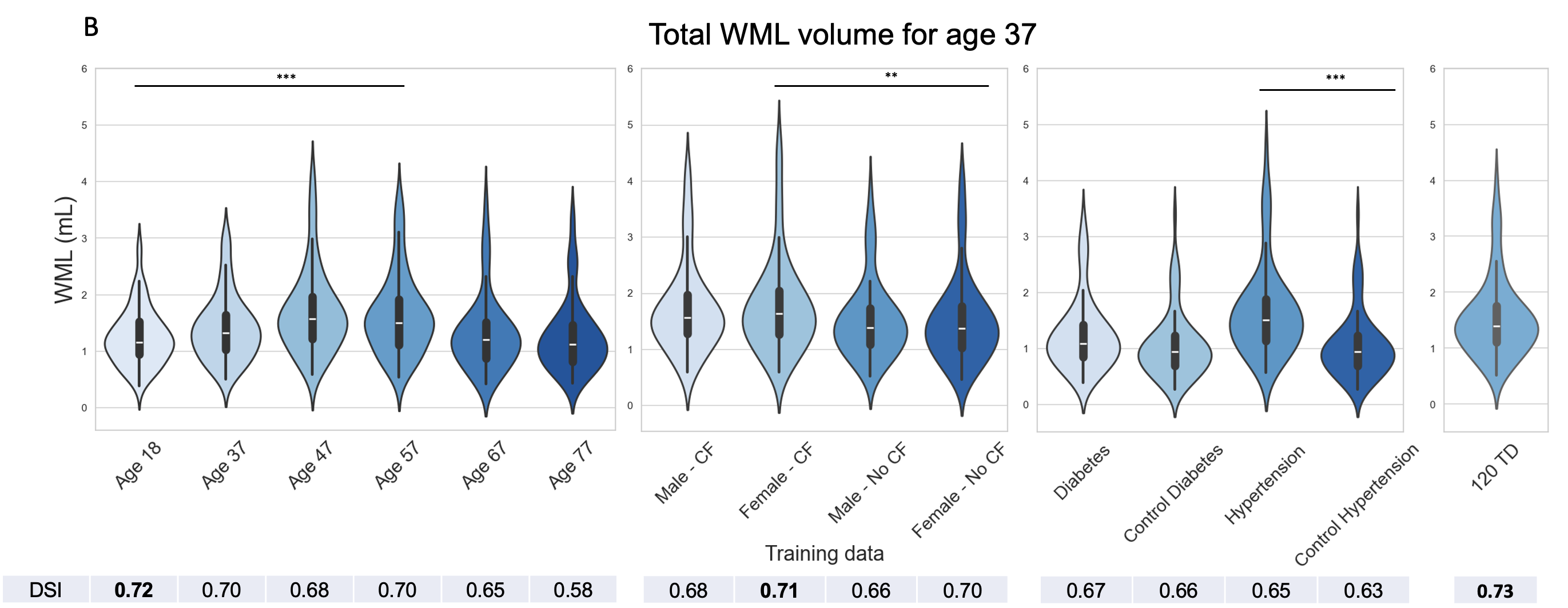


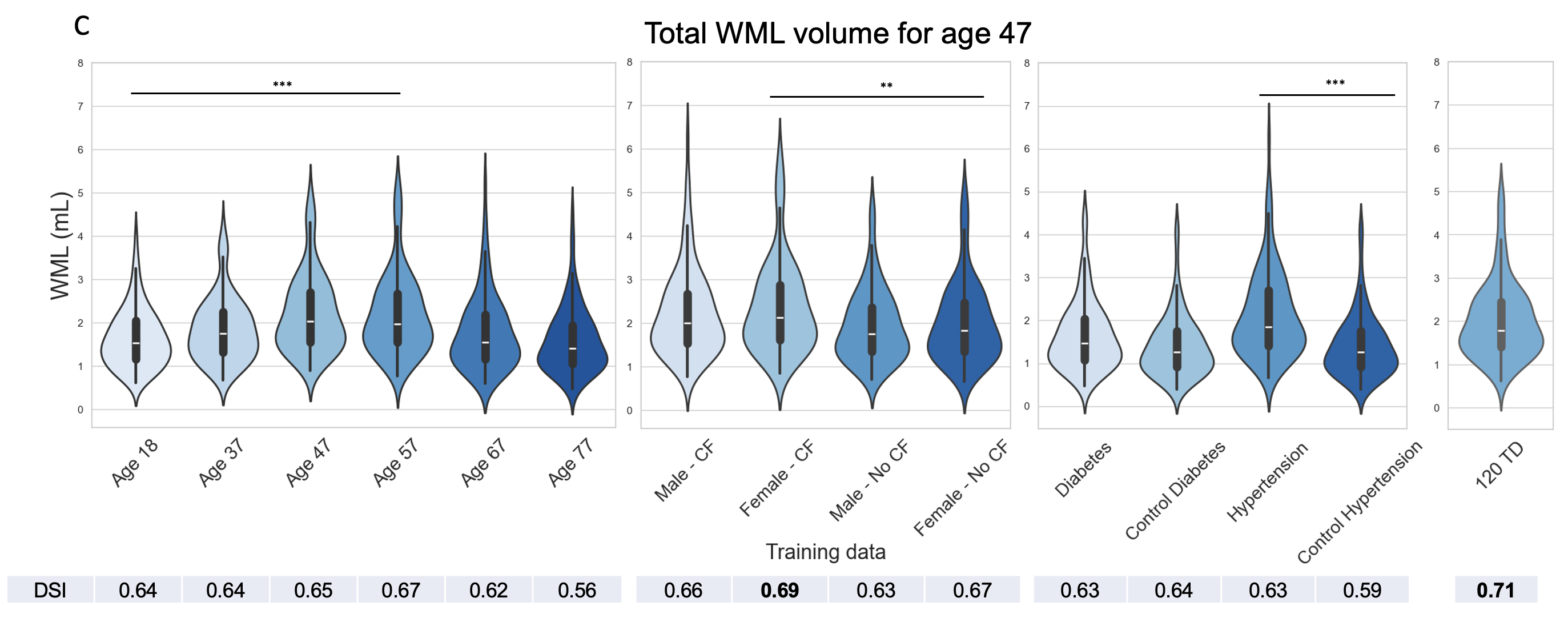


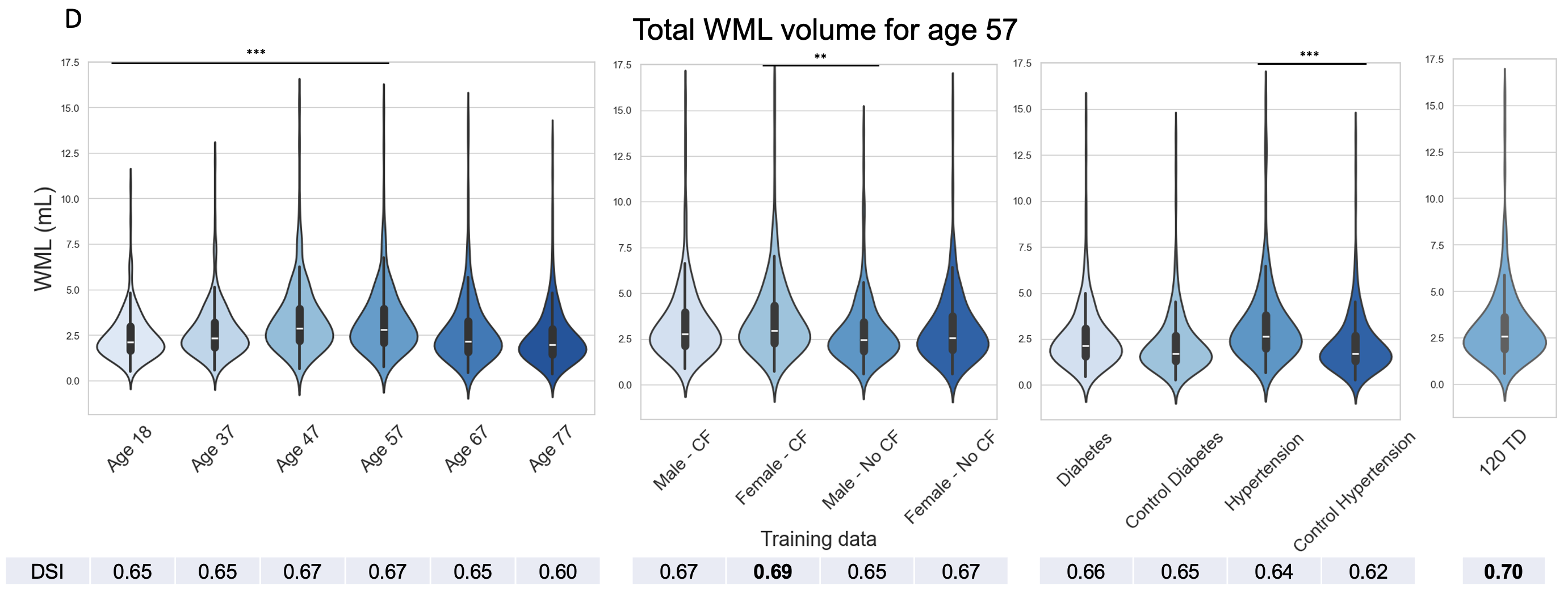


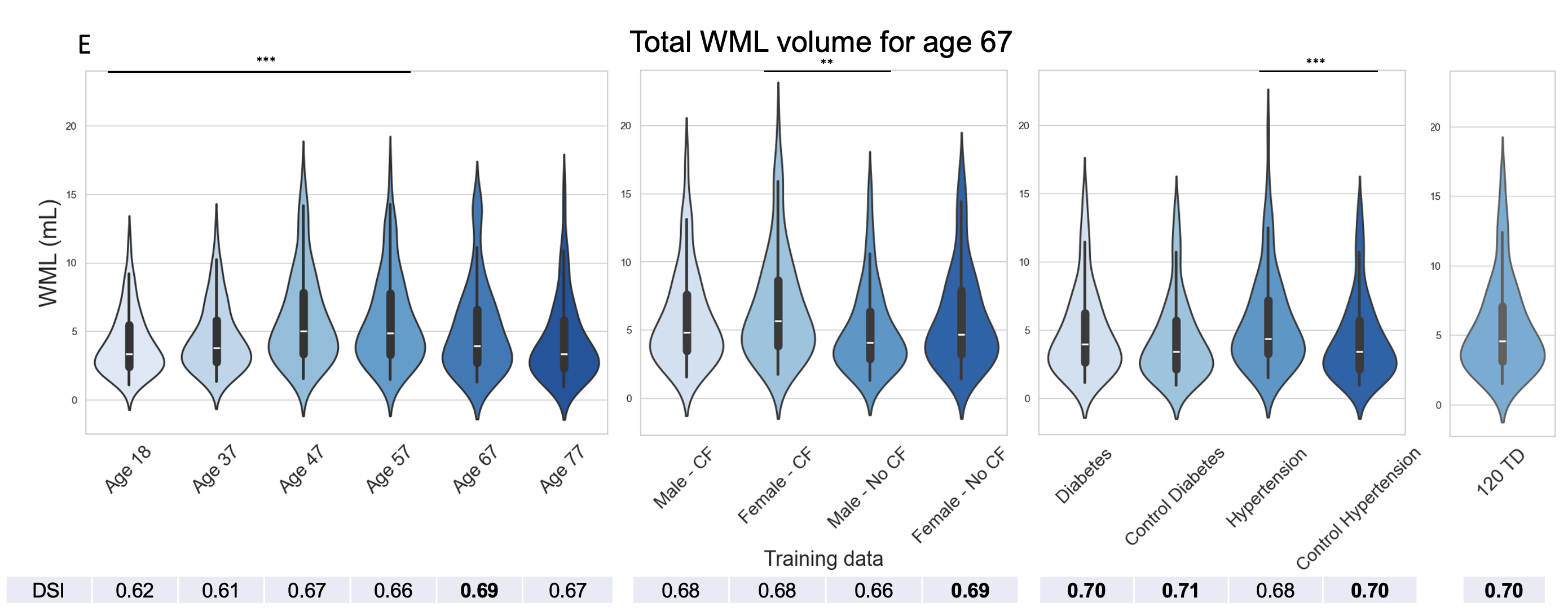


*Fig. s1: Influence of different training datasets on BIANCA’s WML estimations within age subgroups*

Displays the impact of 15 distinct training datasets (x-axis) on the estimation of total WML volume within five test age subgroups: ‘age 18’ (18-37 years old; A), ‘age 37’ (37-47 years old; B), ‘age 47’ (47-57 years old; C), ‘age 57’ (57-67 years old; D), and ‘age 67’ (age 67-87 years old; E). The violin plots within each panel illustrate the distribution of total WML volume, with median values and quartiles shown for each training dataset utilised. The x-axis represents the 14 different training datasets employed in each algorithm run. The highest DSI values are presented in bold. **: 0.001 < p <= 0.01; ***: 0.0001 < p <= 0.001


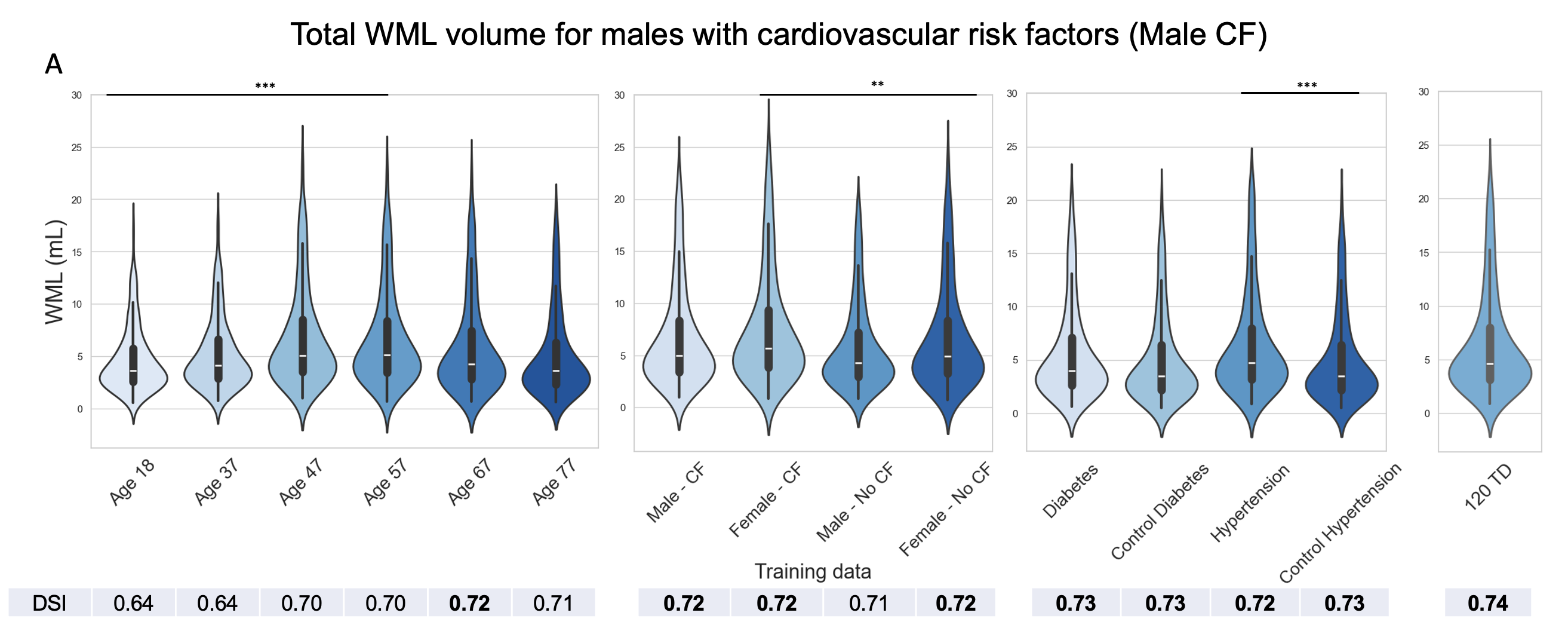


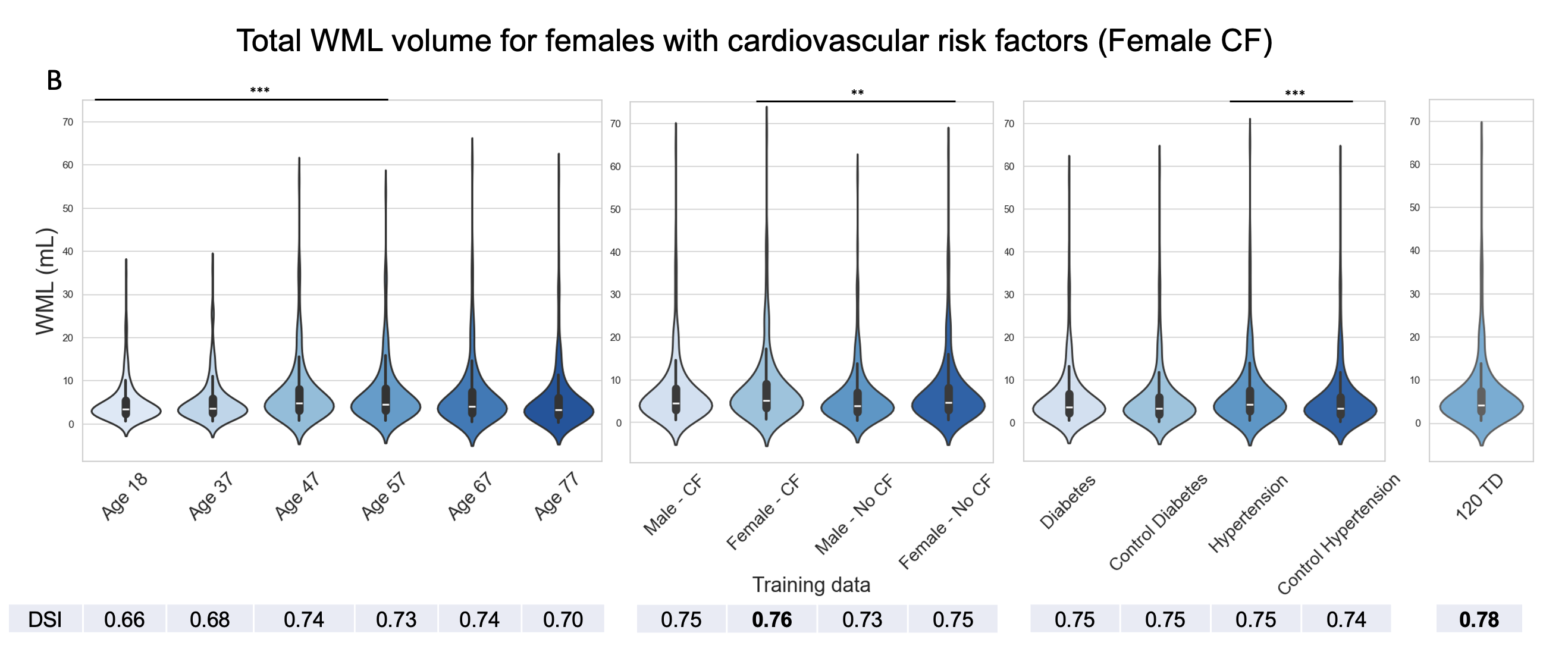


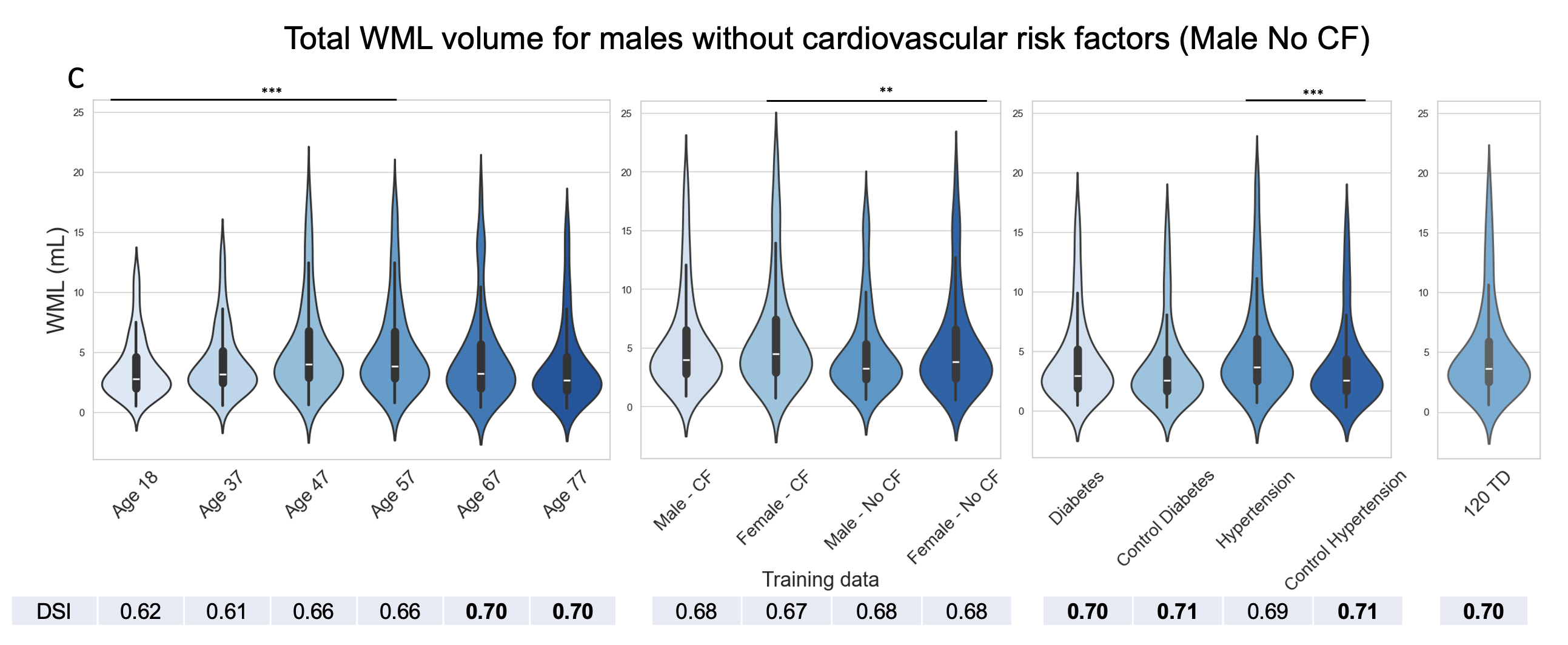


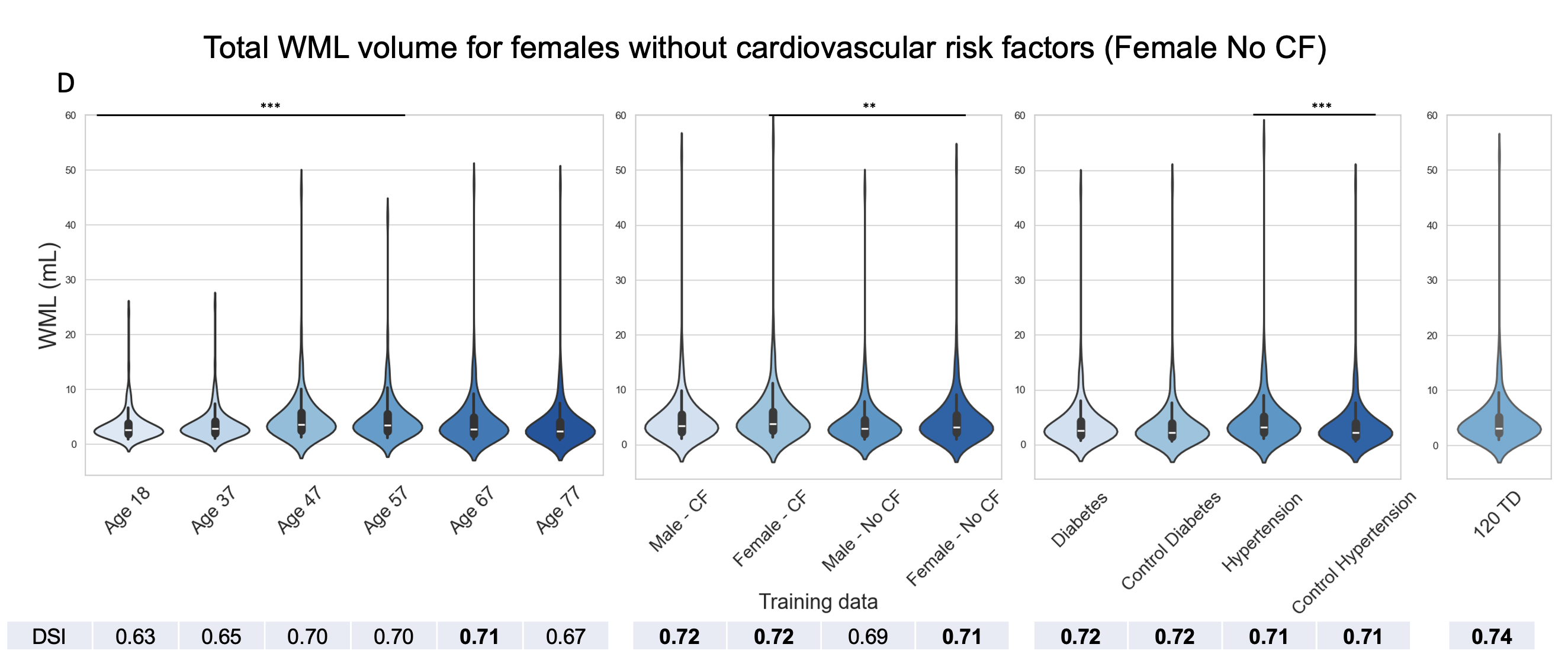


*Fig. s2: Influence of different training datasets on BIANCA’s WML estimations for different sexes*

Influence of 15 distinct training datasets on the WML estimations within four test subgroups defined by sex: Male CF: males with cardiovascular risk factors (A); Female CF: females with cardiovascular risk factors (B); Male no CF: males without cardiovascular risk factors (C); and Female no CF: females without cardiovascular risk factors (D). The x-axis represents the 14 different training datasets. Furthermore, for each run, i.e., for each violin plot, the DSI is indicated with the highest value in bold. **: 0.001 < p <= 0.01; ***: 0.0001 < p <= 0.001


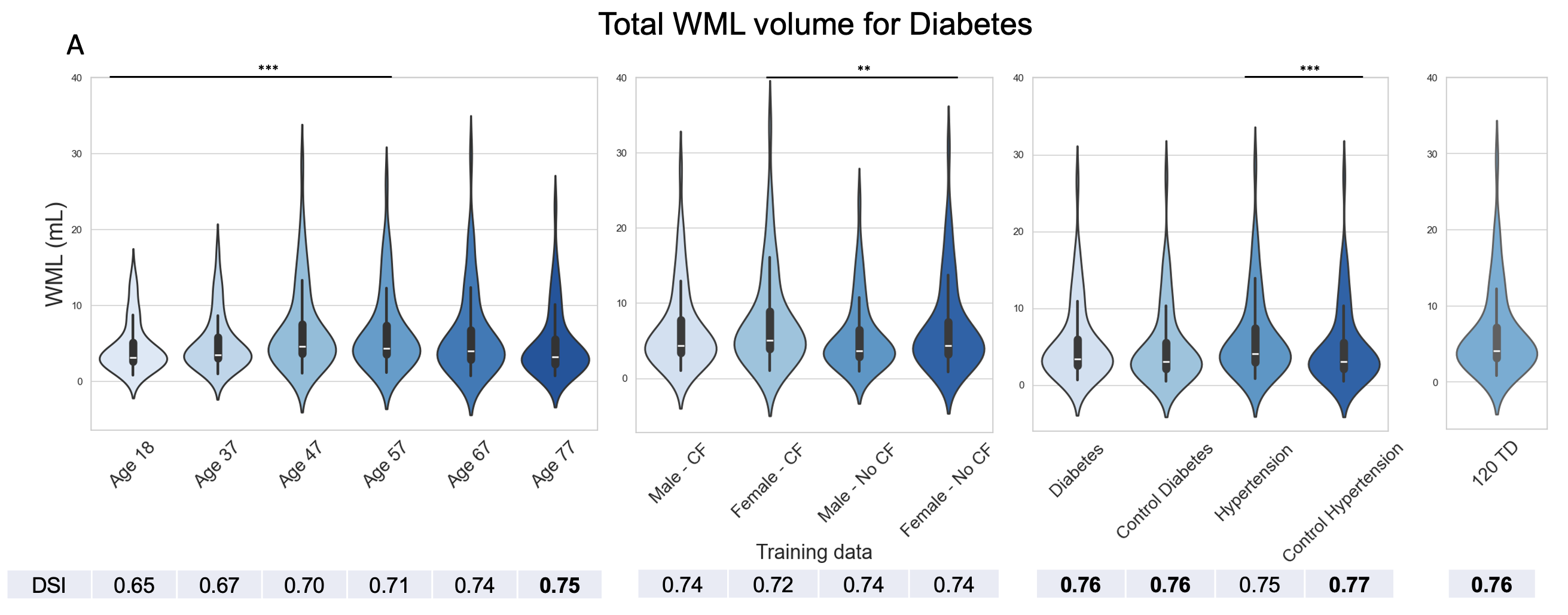


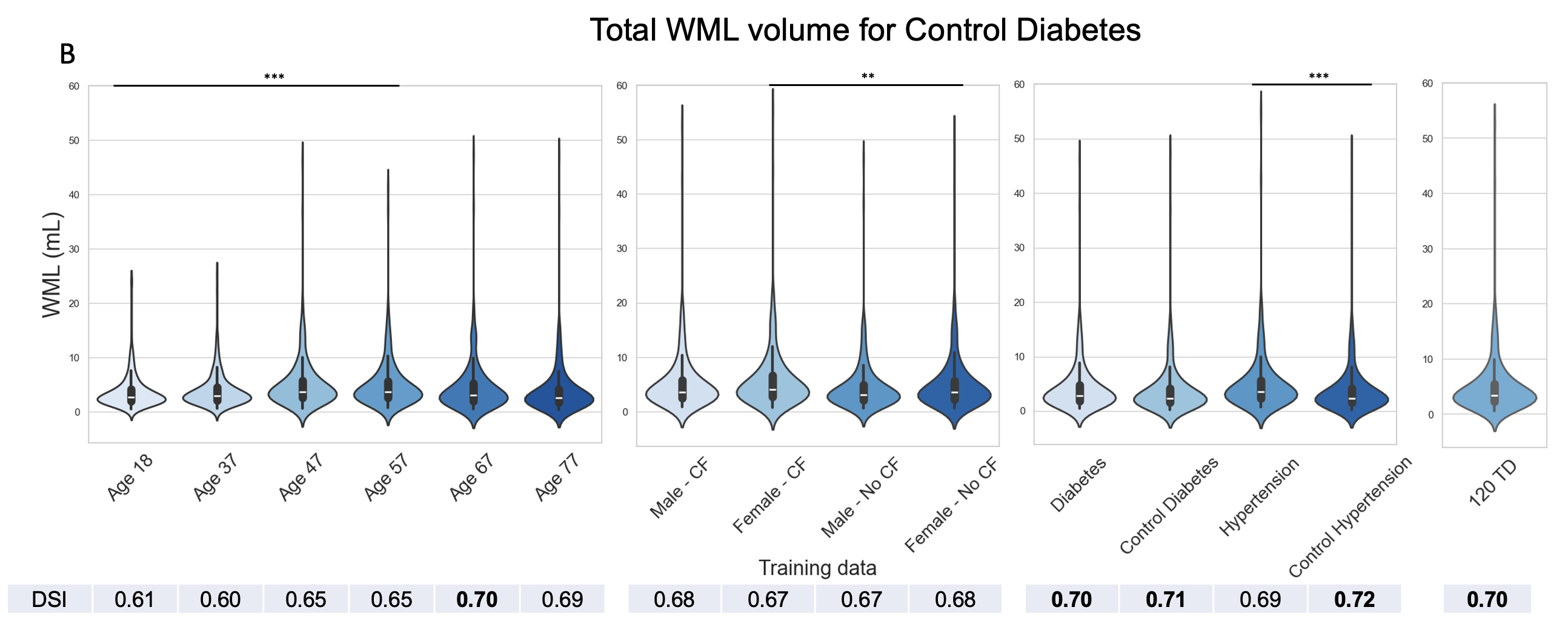


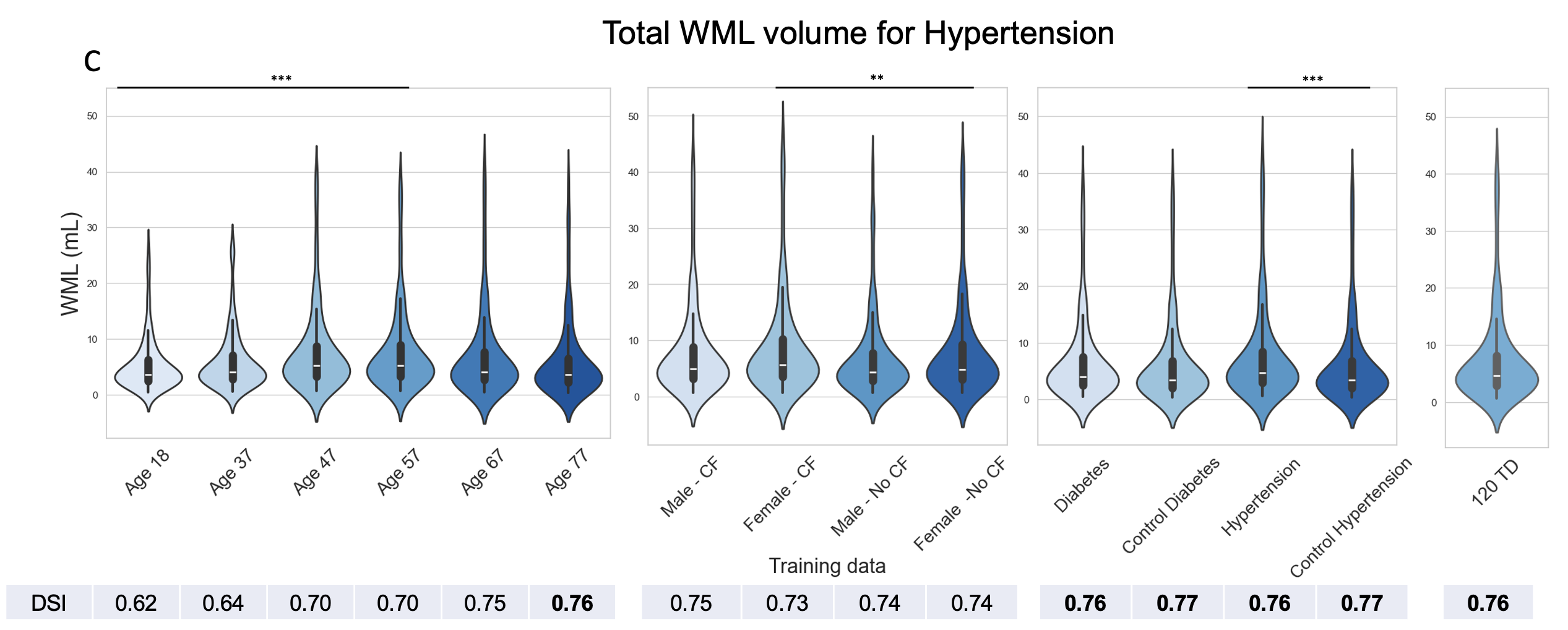


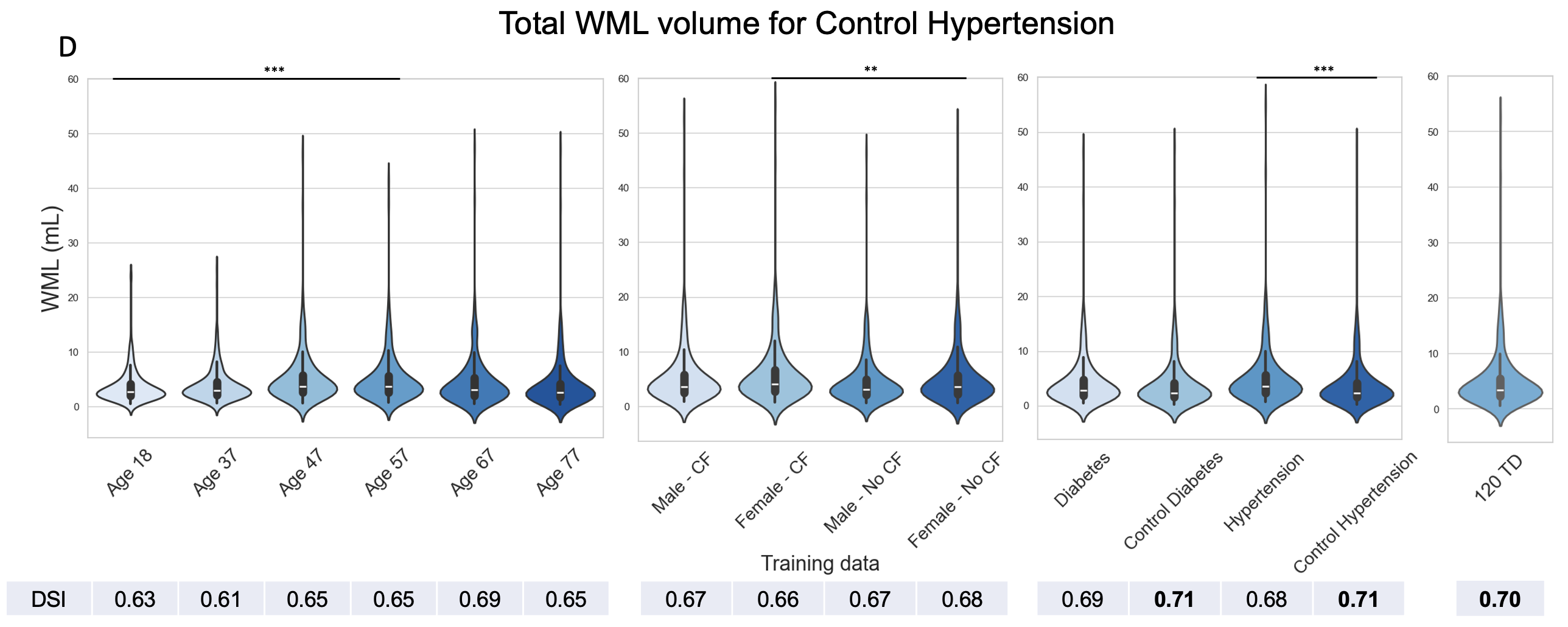


*Fig. s3: Influence of different training datasets on BIANCA’s WML estimations for diabetes, hypertension and control test subgroups*

Displays the impact of 15 distinct training datasets (x-axis) on the estimation of total WML volumes for test ‘diabetes’ (A), ‘control diabetes’ (B), ‘hypertension’ (C), and ‘control hypertension’ (D) subgroups. Furthermore, for each training case, the DSI is depicted with the highest values in bold. **: 0.001 < p <= 0.01; ***: 0.0001 < p <= 0.001
